# Chinese Herbal Medicine for Functional Dyspepsia With Psychological Disorders: A Systematic Review and Meta-Analysis

**DOI:** 10.3389/fnins.2022.933290

**Published:** 2022-07-14

**Authors:** Xiaoying Luo, Lin Wang, Shuangshuang Fang, Xiangli Qing, Tianyuan Jiang, Yang Yang, Xiaolan Su, Wei Wei

**Affiliations:** ^1^Department of Gastroenterology, Wangjing Hospital, China Academy of Chinese Medical Sciences, Beijing, China; ^2^Graduate School of Chengdu University of Traditional Chinese Medicine, Chengdu, China; ^3^Graduate School of Beijing University of Chinese Medicine, Beijing, China

**Keywords:** Chinese herbal medicine, functional dyspepsia (FD), psychological disorder, metaanalysis, effectiveness

## Abstract

**Background and Aims:**

Functional dyspepsia (FD) is closely associated with gut–brain interaction disorder (DGBI), characterized by the interaction of gastrointestinal symptoms and central nervous system dysregulation. Chinese herbal medicine (CHM) has a good concurrent effect in the treatment of FD, especially for patients with concurrent psychological disorders. A meta-analysis was designed to evaluate the efficacy and safety of CHMs in the treatment of FD.

**Methods:**

The PubMed, Embase, Cochrane Library, Web of Science, Chinese Biological Medical Database (CBM), Wanfang Data, China National Knowledge Infrastructure (CNKI), and China Science and Technology Journal Database (VIP) were searched to collect randomized controlled trials of FD treated with CHM. The retrieval time limit is from the establishment of the database till 11 April 2022. Two researchers independently searched databases, screened documents, extracted data, and evaluated the risk of bias of included studies. RevMan 5.4 software was used for meta-analysis.

**Results:**

A total of 11 studies including 951 patients were included. The study was divided into two parts. The first part included 5 clinical trials, including 471 patients. The experimental group was treated only with CHM and the control group was only treated with placebo. The results of first part showed that the total effective rate of CHM in the treatment of FD was higher than that in the placebo group (84.5 vs. 49.4%) [relative risk (RR) = 1.76; 95% confidence interval (CI) (1.13, 2.75); *P* = 0.01]. In addition, CHM treatment could reduce the total symptom score [standardized mean difference (SMD) = −10.05; 95% CI (−13.50, −6.59); *Z* = 5.70; *P* < 0.0001] and depression score [SMD = −7.68; 95% CI (−14.43, −0.94); *Z* = 2.23; *P* = 0.03]. The second part included 6 clinical trials, including 480 patients. The experimental group was only treated with CHM and the control group was treated with prokinetic agents combined with flupentixol melitracen (deanxit). The results of second part showed that the total effective rate of CHM in the treatment of FD was higher than that of the control group (92.6 vs. 78.8%) [RR = 1.17; 95% CI (1.09, 1.26), *P* < 0.0001]. In addition, CHM treatment could reduce HAMA score [mean difference (MD) = −3.19; 95% CI (−3.79, −2.59); *Z* = 10.40; *P* < 0.00001], HAMD score [MD = −4.32; 95% CI (−6.04, −2.61); *Z* = 4.94; *P* < 0.00001], and gastric emptying rate [MD = 12.62; 95% CI (5.84, 19.40); *Z* = 3.65; *P* = 0.0003]. The results of the two parts of the meta-analysis showed no serious adverse reactions, and there was no significant difference in the adverse reactions between the experimental group and the control group [MD = 1.14; 95% CI (0.53, 2.42); *Z* = 0.33; *P* = 0.74]; [MD = 0.14; 95% CI (0.01, 2.67); *Z* = 1.30; *P* = 0.19].

**Conclusion:**

The current evidence shows that CHM treatment has great potential and safety in alleviating the symptoms of FD and improving the psychological disorders of anxiety and depression in patients with FD. Limited by the quantity and quality of the included studies and other biases, the above conclusions need more high-quality studies to be verified.

**Systematic Review Registration:**

https://www.crd.york.ac.uk/PROSPERO/, identifier [CRD42022311129].

## Introduction

Functional dyspepsia (FD) is a common digestive system disease. In 2016, the Rome Committee defined functional gastrointestinal diseases, including FD, as abnormal brain–intestinal interactions (Drossman and Hasler, 2016). The prevalence of FD is about 16% in the general population and up to 18–45% in China (Ge, 2017; Ford et al., 2020). Characteristic symptoms of FD include epigastric pain, epigastric burning, postprandial fullness, or early satiety that persists for at least 6 months. Although the disease does not have obvious organic lesions, the symptoms are persistent and difficult to heal, and easy to repeat, which seriously affects the quality of life and physical and mental health of patients (Ge, 2017).

The pathophysiological mechanism of FD is complex, and its pathogenesis is the result of the combined effects of multiple factors such as gastrointestinal motility disorder, visceral hypersensitivity, intestinal flora imbalance, dysfunction of the gut–brain axis, and mental and emotional factors (Ford et al., 2020). FD is a typical physical and mental disease of the digestive system with a co-morbidity rate of up to 49.3% with psychological disorders (Chen, 2016; Xiong, 2016). With the accelerated pace of life and work, the relationship between FD and psychological factors has been extensively studied. Studies have shown that (Zhu et al., 2015; Zhang, 2018) abnormal emotional factors can lead to brain–gut axis dysfunction, visceral hypersensitivity, and gastrointestinal inflammation and immunity, causing or promoting the occurrence of FD.

The current treatment modalities for FD mainly include pharmacotherapy, lifestyle modification, and psychotherapy (Ford et al., 2020). Clinical medications are generally used for symptomatic treatment, such as acid inhibitors and prokinetics, but these drugs often do not provide complete relief of symptoms. Central neuromodulators have an important role in refractory functional gastrointestinal disease and are especially suitable for patients with combined psychological disorders, but they have many side effects and adverse reactions, and their symptoms tend to worsen after patients stop the antidepressant treatment, so there are many limitations in clinical application. Psychotherapy generally needs to be administered in conjunction with a specialist clinic, and patients who are unable or unwilling to receive treatment are unlikely to benefit. Moreover, a Chinese research study showed that more than 90% of patients with psychological disorders seen in gastroenterology departments were unwilling to receive psychotherapy (Feng et al., 2021). FD is prone to recurring clinically and the treatment effect is not good, which is a considerable burden for individuals and society (Drossman, 2021). Therefore, the search for an effective treatment is a critical issue that needs to be urgently addressed (Ford et al., 2021).

Given the limitations of clinical treatment methods, Chinese herbal medicine (CHM) has shown evident advantages in the treatment of FD. A previous randomized controlled trial (RCT)-based meta-analysis by our team showed that Chinese medicine compounds are more effective than placebo in treating FD due to the improved indigestion symptoms, CMS, gastric emptying rate, and the quality of life of patients with FD (Xiaoying et al., 2021). In recent years, an increasing number of scholars have paid attention to the therapeutic effect of CHM on FD with psychological disorders. Therefore, this study is based on randomized clinical trials. Based on previous studies, the literature has been updated, and more attention has been paid to the improvement of clinical symptoms in FD patients with psychological disorders. The clinical efficacy and safety of CHM in treating FD with psychological disorders were systematically evaluated with the aim of drawing a higher level of evidence and a more objective and a comprehensive evaluation conclusion to guide clinical treatment.

## Data and Methods

### Literature Search

The meta-analysis was conducted according to the Preferred Reporting Items for Systematic Reviews and Meta-Analysis Statement (PRISMA). We searched eight databases including PubMed, Embase, Cochrane Library, Web of Science, China National Knowledge Infrastructure (CNKI), Chinese Biological Medical Database (CBM), Wanfang, and China Science Journal Database (VIP). The retrieval time is from the establishment time of each database to 11 April 2022. The conference papers and dissertations of related clinical trials were simultaneously retrieved in CNKI and Wanfang data resource systems.

The following search terms were used: (functional dyspepsia OR postprandial distress syndrome OR epigastric pain syndrome) AND (psychological disorders OR anxiety OR depression) AND (Traditional Chinese Medicine OR Chinese Medicine OR Chinese Traditional Medicine OR Herbal Medicine OR formula OR Decoction OR recipe OR prescription OR tablet OR capsule OR granule) AND (random).

### Study Selection

#### Inclusion Criteria

##### Study Type

Clinical RCTs published in Chinese or English.

##### Research Objects

The participants were patients with FD over the age of 18 who met the diagnostic criteria of Rome II, Rome III, or Rome IV and at least have 1 psychological disorder (Talley et al., 1999; Tack et al., 2006; Stanghellini et al., 2016). Patients with severe organic or mental diseases were excluded from the study.

##### Intervention Measures

①Treatment group: only oral CHM treatment and does not involve the addition and subtraction of drug taste and dosage; there is no limit to the form of CHM (decoction, granules, capsules, etc.), and the course of medication should not be less than 2 weeks.
②Control group: the first part is only oral placebo control. The placebo should conform to the shape, nature, and taste of CHM similar to that of the treatment group, and the course of treatment is the same as that of the treatment group. The second part is the combination of prokinetics + deanxit.

##### Curative Effect Evaluation Index

Main outcome evaluation index:

①The total effective rate with the extractable dichotomous variable data. A patient-reported assessment is preferred if the study involved both investigator-reported and patient-reported results.
②The scores of the scale reflecting psychological disorders such as HAMD, HAMA, SDS, SAS, and so on.

Secondary outcome evaluation indexes: total symptom score, gastric emptying rate, and adverse reactions.

If the main outcome evaluation index does not meet the above requirements, the outcome evaluation index, including TCM syndrome efficacy or gastric emptying rate can also be included; studies reporting different outcome indicators from the same clinical trial were combined and included.

#### Exclusion Criteria

(1) Non-English and Chinese literature; (2) the original information is not published publicly; (3) interventions are interfered by other treatments; (4) unable to get the full text or the data is incomplete; and (5) repeated published literature.

### Data Extraction

Two researchers independently read the title, abstract, and full text of the literature and screened and included the literature according to the standard of arrangement. In the case of objection, the third party intervened to evaluate it. The extracted data included literature title, author, publication date, research source, sample size, western medicine diagnostic criteria, intervention measures, course of treatment and follow-up time, outcome evaluation index and results, and adverse reaction events.

### The Quality Evaluation of Included Literature

Two researchers independently conducted the quality evaluation, and a third investigator evaluated the objections. This evaluation was performed according to the Cochrane ROB Tool in terms of: generation of random sequence, concealment of allocation, blind method, incomplete outcome data, selective reporting, and other biases, and the results were exported *via* Review Manager V 5.4.

### Statistical Analysis

We used Rev Man 5.4 for merge-effect analysis. Two classification variables were analyzed by relative risk (RR), and the numerical variables were analyzed by mean difference (MD) or standardized mean difference (SMD), all of which were expressed by 95% confidence intervals (CIs).

The heterogeneity among statistics of multiple identical studies was tested by tests for heterogeneity and evaluated using the *X*^2^ test combined with *I*^2^ statistics. If *P* > 0.10 and *I*^2^ < 50%, the heterogeneity was considered acceptable (Higgins et al., 2003). If the included research has homogeneity, the fixed effects model is used, and if there is heterogeneity, a random-effect model (Dersimonian and Laird, 1986) is used. It was considered to be statistically significant when *P* < 0.05.

## Results

### Retrieval Results

**FIGURE 1:**
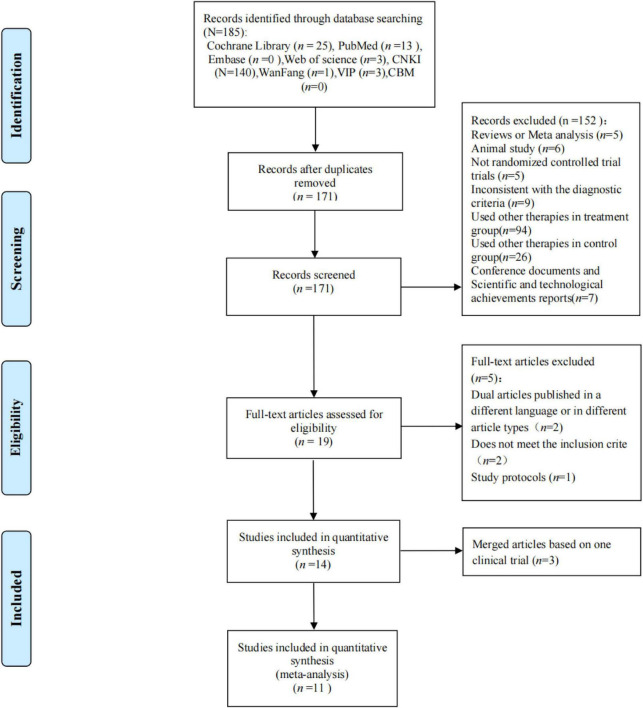
Article screening process.

A total of 185 studies were obtained through preliminary search, including 140 in CNKI, 1 in Wanfang, 13 in PubMed, 3 in VIP, 25 in Cochrane, and 3 in Web of Science. First, we removed 14 duplicate documents. Second, 152 articles were excluded by reading the literature titles and abstracts, and 8 articles were excluded by reading the full text. Finally, 11 studies were included in the meta-analysis (Zhao and Gan, 2005; Han and Wang, 2011; Lu and Chen, 2011; Du et al., 2014; Xi et al., 2014; Zhang, 2014; Zhang et al., 2016, 2017a,b,c; Zhu and Gu, 2017; Li et al., 2018; Tominaga et al., 2018; Chen et al., 2020). Among them, there are five studies (Zhao and Gan, 2005; Du et al., 2014; Zhu and Gu, 2017; Tominaga et al., 2018; Chen et al., 2020) on CHM vs. placebo and six studies (Han and Wang, 2011; Lu and Chen, 2011; Xi et al., 2014; Zhang, 2014; Zhang et al., 2016, 2017a,b,c; Li et al., 2018) on CHM vs. prokinetics + deanxit (Figure 1).

### Basic Characteristics of Included Studies

A total of 11 RCTs were selected. Among them, there are five studies on CHM vs. placebo, one (Tominaga et al., 2018) from Japan and the rest (Zhao and Gan, 2005; Du et al., 2014; Zhu and Gu, 2017; Chen et al., 2020) from China. Four (Zhao and Gan, 2005; Du et al., 2014; Tominaga et al., 2018; Chen et al., 2020) of all studies were published in English and one (Zhu and Gu, 2017) in Chinese.

There are six studies of CHM vs. prokinetics + deanxit, all from China and published in Chinese. The 951 participants in the 11 RCTs were divided into the following 2-part studies, and the number of participants in each study ranged from 43 to 118. Of the 11 studies involved, 2 (Tominaga et al., 2018; Chen et al., 2020) were multicenter studies, and the number of participating research centers ranged from 9 to 56. The basic information of the 11 studies included in the analysis is shown in Table 1. The specific drug composition of Chinese medicine compounds involved in the study is shown in Table 2.

**TABLE 1:** Basic characteristics of included articles.

References	Language	Country	Diagnostic criteria	Psychological disorders	Number of research centers	Sample size (T:C)	Sex ratio (male: female)	Durations	Follow-up	Outcomes	Adverse events (T:C)
**CHM vs. placebo**											
Zhao and Gan, 2005	English	China	Rome III	HAMD > 20; HAMA > 14	1	43 (30:13)	43 (16:27)	8 weeks	NR	①②③④	0
Du et al., 2014	English	China	Rome III	Depression	1	180 (90:90)	NR	8 weeks	6 months	①③⑤	0
Tominaga et al., 2018	English	Japan	Rome III	HADS < 10	56	118 (61:57)	125 (36:89)	8 weeks	NR	①②③	4 (3:1)
Chen et al., 2020	English	China	Rome III	HAMD > 20; HAMA > 14	9	141 (70:71)	141 (107:3)	4 weeks	4 weeks	②③④	4 (3:1)
Zhu and Gu’s (2017)	Chinese	China	Rome III	Mild to moderate depression on the HAMD-17	1	80 (48:32)	80 (37:43)	6 weeks	NR	②③	15 (8:7)
**CHM vs. mosapride/domperidone + deanxit**											
Han and Wang, 2011	Chinese	China	Rome III	HAMD ≥ 17	1	60 (30:30)	60 (20:40)	4 weeks	NR	①③	NR
Lu and Chen, 2011	Chinese	China	Rome III	HAMA > 7; HAMD > 7	1	55 (31:24)	55 (19:36)	4 weeks	NR	①③④	NR
Xi et al., 2014	Chinese	China	Rome III	HAMD ≥ 8	1	96 (48:48)	96 (39:57)	30 days	6 months	①③⑤	0
Zhang, 2014	Chinese	China	Rome III	HAMD ≥ 7	1	70 (35:35)	70 (29:41)	30 days	3 months	①③⑤	3 (0:3)
Zhang et al., 2016, 2017a,b,c	Chinese	China	Rome III	HAMD > 20; HAMA > 14	1	119 (60:59)	119 (49:70)	4 weeks	NR	①③④	Incomplete information
Li et al., 2018	Chinese	China	Rome III	HAMD ≥ 20; HAMA ≥ 14	1	80 (40:40)	80 (34:46)	4 weeks	NR	①③④⑤	0

*T, treatment group; C, control group; NR, not report. ① Total efficiency, ② total symptom score, ③ depression scale, ④ HAMA score, and ⑤ gastric emptying rate.*

**TABLE 2:** Composition of Chinese medicine compounds.

References	Chinese herbal medicine	Control group	Chinese herbal formula
**CHM vs. placebo**			
Zhao and Gan, 2005	Xinwei decoction (1 dose, TID)	Placebo (1 dose, TID)	Chaihu (Radix Bupleuri) 10 g, Xiangfu (Rhizoma Cyperi) 10 g, Hehuanhua (Flos Albiziae) 30 g, Meiguihua (Flos Rosae Rugosae) 20 g, Taizishen (Radix Pseudostellariae) 15 g, Quangualou (Fructus Trichosanthis) 15 g, Baizhu (Rhizoma Atractylodis Macrocephalae) 10 g, Zhishi (Fructus Aurantii Immaturus) 10 g, Sharen (Fructus Amomi) 10 g, Yujin (Radix Curcumae) 12 g, Fushen (Sclaerotium Poriae Circum Radicem Pini) 15 g, Baihe (Bulbus Lilii) 15 g, Xiangyuan (Fructus Citri) 10 g, parched Maiya (Fructus Hordei Germinatus) 10 g, parched Guya (Fructus Oryzae Germinatus) 10 g, Qiancaogen (Radix Rubiae) 12 g, and Xuchangqing (Radix Cynanchi Paniculati) 15 g
Du et al., 2014	Xiaoyao pill (3 g, BID)	Placebo (3 g, BID)	Chai Hu (radix bupleuri), Dang Gui (*Angelica sinensis*), Bai Shao (radix paeoniae alba), Chao Bai Zhu (roasted rhizoma atractylodis macrocephalae), Fu Ling (*Wolfiporia extensa*), Zhi Gan Cao (radix glycyrrhizae), Bo He (mint), and Sheng Jiang (rhizoma zinjiberis recens)
Tominaga et al., 2018	Rikkunshito (7.5 g, TID)	Placebo (7.5 g, TID)	NR
Chen et al., 2020	Formulation of Jiawei Xiaoyao (6 g, BID)	Placebo (6 g, BID)	NR
Zhu and Gu’s (2017)	Morinda officinalis oligose capsule (1 pill, BID)	Placebo (1 pill, BID)	NR
**CHM vs. mosapride/domperidone + deanxit group**			
Li et al., 2018	Danzhi Xiaoyao San and Simo Soup and Simo decoction (1 dose, BID)	Mosapride citrate tablets (5 mg, TID); deanxit (1 pill, BID)	Muxiang (AUCKLANDIAE RADIX) 10 g, Wuyao (LINDERAE RADIX) 10 g, Zhiqiao (AURANTII FRUCTUS) 10 g, Binglang (ARECAE SEMEN) 10 g, Mudanpi (MOUTAN CORTEX) 10 g, Zhizi (GARDENIAE FRUCTUS) 10 g, Chaihu (BUPLEURI RADIX) 10 g, Fuling (PORIA) 10 g, Danggui (ANGELICAE SINENSIS RADIX) 10 g, Baishao (PAEONIAE RADIX ALBA) 15 g, Baizhu (RHIZOMA ATRACTYLODIS) 10 g, Gancao (GLYCYRRHIZAE RADIX ET RHIZOMA) 5 g
Xi et al., 2014	Modified Sini Powder (1 dose, BID)	Domperidone maleate tablets (12.72 mg, TID); deanxit (1 pill, BID)	Fushen (PORIA) 20 g, Chaihu (BUPLEURI RADIX) 12 g, Zhishi (AURANTII FRUCTUS IMMATURUS) 12 g, Baizhu (RHIZOMA ATRACTYLODIS) 10 g, Baishao (PAEONIAE RADIX ALBA) 10 g, Chenpi (CITRI RETICULATAE PERICARPIUM) 12 g, Dafupi (ARECAE PERICARPIUM) 20 g, Shichangpu (ACORI TATARINOWII RHIZOMA) 10 g, Yujin (CURCUMAE RADIX) 12 g, Gancao (GLYCYRRHIZAE RADIX ET RHIZOMA) 6 g
Zhang, 2014	Recipe of soothing the liver and regulating the stomach (200 ml, BID)	Domperidone maleate tablets (12.72 mg, TID); deanxit (1 pill, BID)	Fushen (PORIA) 20 g, Chaihu (BUPLEURI RADIX) 12 g, Baizhu (RHIZOMA ATRACTYLODIS) 18 g, Zhishi (AURANTII FRUCTUS IMMATURUS) 12 g, Dafupi (ARECAE PERICARPIUM) 20 g, Chenpi (CITRI RETICULATAE PERICARPIUM) 12 g, Baishao (PAEONIAE RADIX ALBA) 10 g, Yujin (CURCUMAE RADIX) 12 g, Shichangpu (ACORI TATARINOWII RHIZOMA) 10 g, Gancao (GLYCYRRHIZAE RADIX ET RHIZOMA) 6 g
Zhang et al., 2016; Zhang et al., 2017a,b,c	Shugan Jianpi Anshen recipe (100 ml, BID)	Domperidone tablets (10 mg, TID); deanxit (10 mg, BID)	Chaihu (BUPLEURI RADIX) 10 g, Shichangpu (ACORI TATARINOWII RHIZOMA) 10 g, Ezhu (CURCUMAE RHIZOMA) 10 g, Huanglian (COPTIDIS RHIZOMA) 10 g, Houpo (MAGNOLIAE OFFICINALIS CORTEX) 12 g, Zhiqiao (AURANTII FRUCTUS) 10 g, Yujin (CURCUMAE RADIX) 12 g, Qingbanxia (PINELLIAE RHIZOMA PRAEPARATUM CUM ALUMINE) 15 g, Baishao (PAEONIAE RADIX ALBA) 10 g, Baizhu (RHIZOMA ATRACTYLODIS) 15 g, Fuling (PORIA) 15 g, Baihe (LILII BULBUS) 15 g, Shanzha (CRATAEGI FRUCTUS) 15 g, Maiya (HORDEI FRUCTUS GERMINATUS) 15 g, Shenqu (MEDICATED LEAVEN) 15 g, Hehuanpi (ALBIZIAE CORTEX) 20 g
Han and Wang, 2011	Modified Sini Powder (1 dose, BID)	Domperidone tablets (10 mg, TID); deanxit (10.5 mg, QD)	Chaihu (BUPLEURI RADIX) 6 g, Zhiqiao (AURANTII FRUCTUS) 10 g, Baishao (PAEONIAE RADIX ALBA) 12 g, Zhigancao (GLYCYRRHIZAE RADIX ET RHIZOMA PRAEPARATA CUM MELLE) 6 g, Lianqiao (FORSYTHIAE FRUCTUS) 10 g, Hehuanpi (ALBIZIAE CORTEX) 12 g, Xiangfu (CYPERI RHIZOMA) 6 g, Muxiang (AUCKLANDIAE RADIX) 6 g, Qingbanxia (PINELLIAE RHIZOMA PRAEPARATUM CUM ALUMINE) 9 g

*NR, no report.*

### Risk of Bias Assessment

**FIGURE 2:**
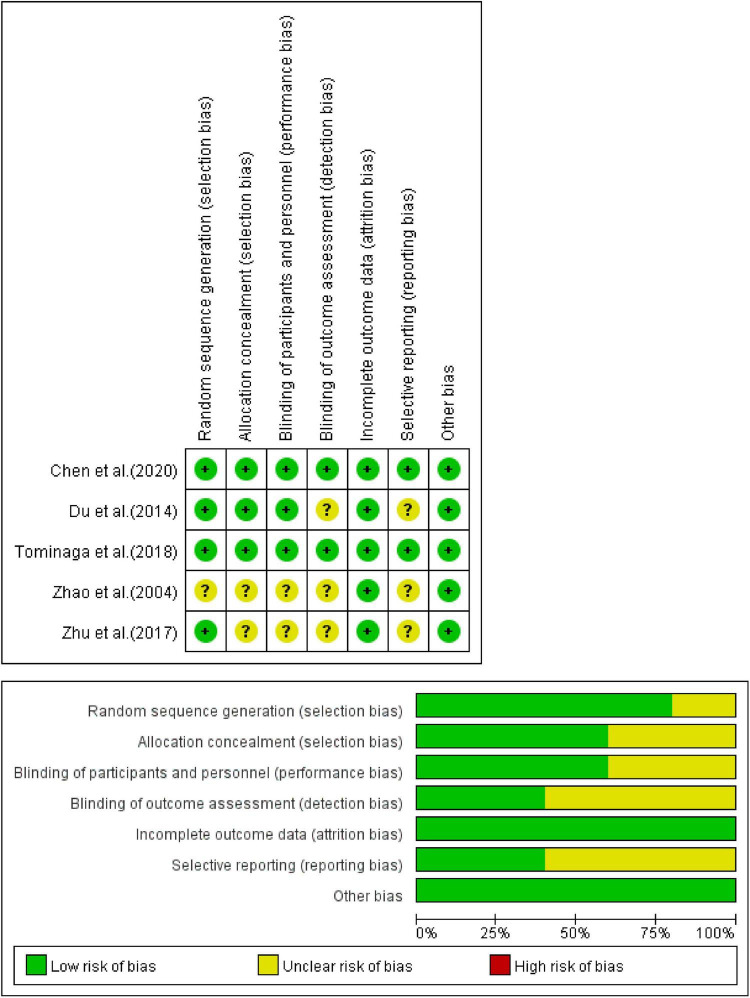
Assessment of the risk of bias of CHM vs. placebo.

**FIGURE 3:**
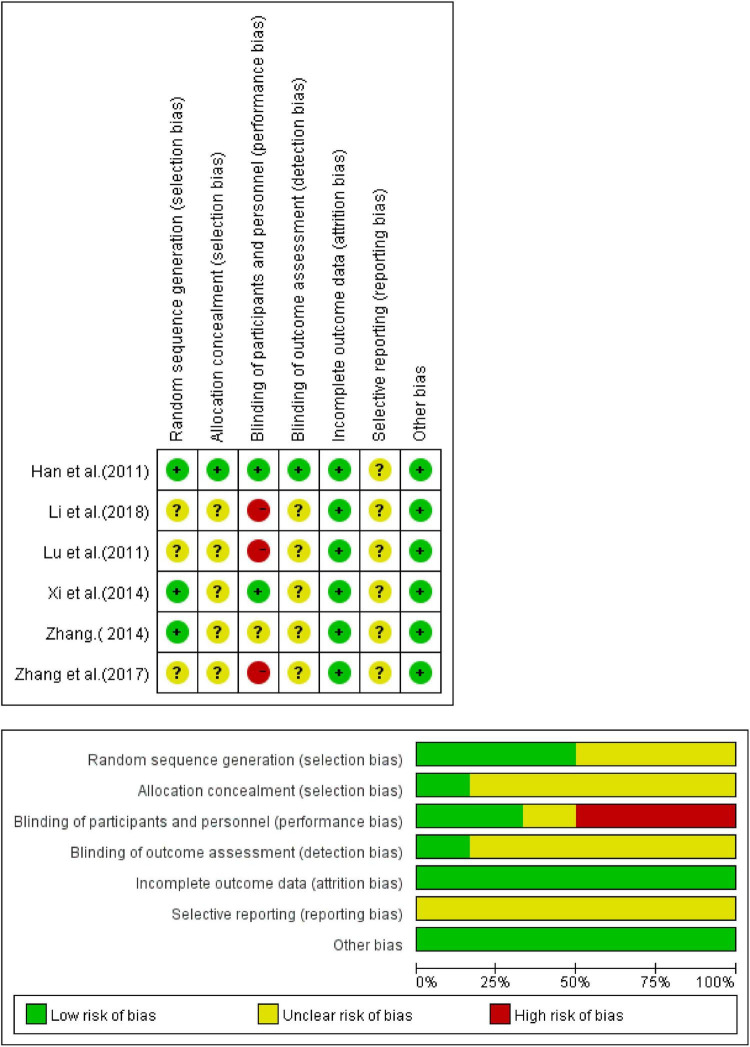
Assessment of the risk of bias of CHM vs. western medicine group.

All the inclusion trials were randomized, but some of them did not describe the specific randomization method, hence the evaluation was “unclear.” Some of these inclusion trials did not describe blindness. Among them, trials that neither described randomization method nor blindness were considered high risk, and the others were evaluated as “unclear.” Complete details of the bias risk assessment are shown in Figures 2, 3.

### Meta Results

#### Total Effective Rate

##### Chinese Herbal Medicine vs. Placebo Group

Among the five studies included, three studies (Zhao and Gan, 2005; Du et al., 2014; Tominaga et al., 2018) reported the total effective rate. There were a total of 341 cases, of which 181 cases were in the Chinese medicine compound treatment group. Of these, 153 cases were reported effective (84.5%). Of the 160 cases in the placebo control group, of which 79 cases were effective (49.4%).

**FIGURE 4:**
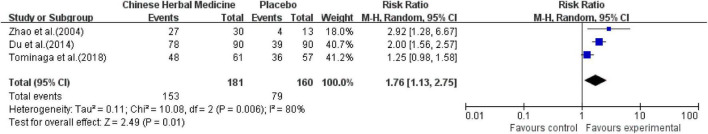
Meta-analysis of total effective rate of CHM vs. placebo.

The heterogeneity test showed high heterogeneity (*P* = 0.006, *I*^2^ = 80%); therefore, a randomized effect model was adopted. The results showed that the therapeutic effect of CHM was significantly better than that of placebo on the overall symptom improvement [RR = 1.76; 95% confidence interval (CI) (1.13, 2.75), *P* = 0.01] (Figure 4).

##### Chinese Herbal Medicine vs. Western Medicine Group

Six studies (Han and Wang, 2011; Lu and Chen, 2011; Xi et al., 2014; Zhang, 2014; Zhang et al., 2016, 2017a,b,c; Li et al., 2018) all reported the total clinical effective rate. Totaling 480 cases, 244 cases were in the Chinese medicine compound treatment group. Of these, 226 cases were effective (92.6%), and of the 236 cases in the control group, 186 cases were effective (78.8%).

**FIGURE 5:**
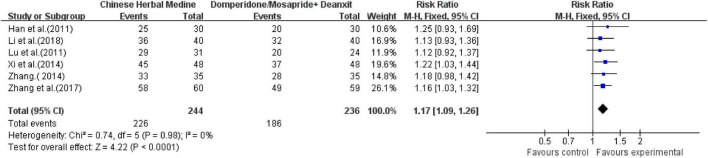
Meta-analysis of total effective rate of CHM vs. western medicine group.

The comprehensive analysis showed low heterogeneity (*I*^2^ = 0, *P* = 0.98), and a fixed-effects model was used. The comprehensive results showed that the effective rate of the CHM was significantly better than that of the control group, and the difference was statistically significant [RR = 1.17; 95% CI (1.09, 1.26), *P* < 0.0001] (Figure 5).

#### Total Symptom Score

##### Chinese Herbal Medicine vs. Placebo Group

**FIGURE 6:**
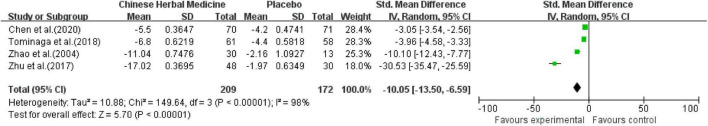
Meta-analysis of total symptom score of CHM and placebo.

A total of 4 studies (Zhao and Gan, 2005; Zhu and Gu, 2017; Tominaga et al., 2018; Chen et al., 2020), including 381 participants compared total symptom scores. The comprehensive analysis showed high heterogeneity (*I*^2^ = 98%), and a random-effects model was used. The comprehensive results showed that the CHM could effectively reduce the total symptom score [SMD = −10.05; 95% CI (−13.50, −6.59); *Z* = 5.70; *P* < 0.00001] (Figure 6).

#### Depression Scale

##### Chinese Herbal Medicine vs. Placebo Group

**FIGURE 7:**
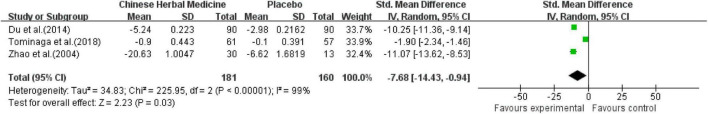
Meta-analysis of depression score of CHM vs. placebo.

Three studies (Zhao and Gan, 2005; Du et al., 2014; Tominaga et al., 2018) including 341 investigators compared post-treatment depression scores. The heterogeneity test showed high heterogeneity (*P* < 0.00001, *I*^2^ = 99%); therefore, a randomized effect model was adopted. Two of the studies used the HAMD score (Zhao and Gan, 2005; Du et al., 2014) and one (Tominaga et al., 2018) study used the HADS score. The comprehensive results showed that the CHM could effectively reduce the depression score [SMD = −7.68; 95% CI (−14.43, −0.94); *Z* = 2.23; *P* = 0.03] (Figure 7).

Two other studies also evaluated depression improvement. According to Chen et al.’s (2020) results, the JX pill group had a greater improvement in the HAMD total score from baseline to 4 weeks than the placebo group, but the difference was not significant [mean between-group difference, −0.7 points (95% CI, −1.8 to −0.3); *P* = 0.093]. Zhu and Gu (2017) showed that compared with the placebo group, the effective rate of the CHM group was significantly increased (68.75 vs. 37.50%, *P* < 0.05). The cure rates of the CHM group were significantly higher than those of the placebo group (27.08% vs. 0, *P* < 0.05).

##### Chinese Herbal Medicine vs. Western Medicine Group

**FIGURE 8:**
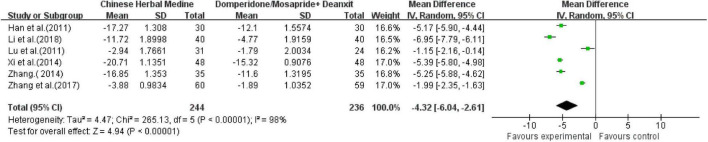
Meta-analysis of HAMD score of CHM vs. western medicine group.

Six studies (Han and Wang, 2011; Lu and Chen, 2011; Xi et al., 2014; Zhang, 2014; Zhang et al., 2016, 2017a,b,c; Li et al., 2018) including 480 investigators compared HAMD scores after treatment. The comprehensive analysis showed high heterogeneity (*I*^2^ = 98%), and a random-effects model was used. The comprehensive results showed that the CHM could effectively reduce the HAMD score [MD = −4.32; 95% CI (−6.04, −2.61); *Z* = 4.94; *P* < 0.00001] (Figure 8).

#### HAMA Score

##### Chinese Herbal Medicine vs. Placebo Group

Only Chen et al. (2020) evaluated anxiety. The results showed that JX pill had a greater improvement in the HAMA scores from baseline to 4 weeks than the placebo group, but the difference was not significant [mean between-group difference, −0.3 points (95% CI, −1.3 to −0.7); *P* = 0.446].

##### Chinese Herbal Medicine vs. Western Medicine Group

**FIGURE 9:**
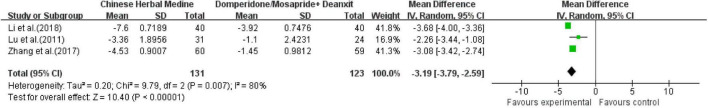
Meta-analysis of HAMA score of CHM vs. western medicine group.

Three studies (Lu and Chen, 2011; Zhang et al., 2016, 2017a,b,c; Li et al., 2018) including 254 investigators compared HAMA scores after treatment. The comprehensive analysis showed high heterogeneity (*I*^2^ = 80%), and a random-effects model was used. The comprehensive results showed that the CHM could effectively reduce the HAMA score [MD = −3.19; 95% CI (−3.79, −2.59); *Z* = 10.40; *P* < 0.00001] (Figure 9).

#### Gastric Emptying Rate

##### Chinese Herbal Medicine vs. Western Medicine Group

**FIGURE 10:**
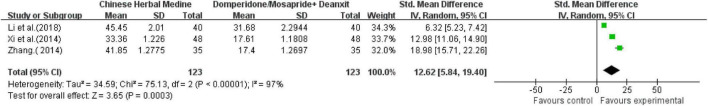
Meta-analysis of gastric emptying rate of CHM vs. western medicine group.

Three studies (Xi et al., 2014; Zhang, 2014; Li et al., 2018) involving six articles reported the gastric emptying rate. All studies used radioimaging. The heterogeneity test showed high heterogeneity (*P* < 0.00001, *I*^2^ = 97%); thus, the randomized effect model was adopted. The combined results showed that the Chinese herbal formula could effectively reduce the gastric emptying rate [MD = 12.62; 95% CI (5.84, 19.40); *Z* = 3.65; *P* = 0.0003] (Figure 10).

#### Adverse Reactions

##### Chinese Herbal Medicine vs. Placebo Group

**FIGURE 11:**
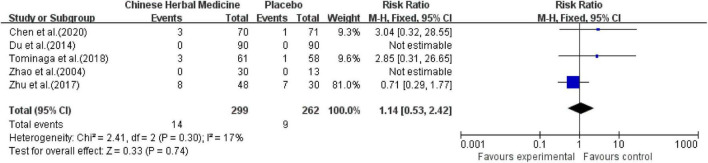
Meta-analysis of adverse reactions of CHM and placebo.

All 5 studies reported drug safety evaluations and included a total of 561 patients. In Zhu and Gu’s (2017) study, eight cases of adverse reactions occurred in the GHM group, including three cases of dry mouth, two cases of dizziness, one case of loss of appetite, one case of nausea, and one case of gastrointestinal discomfort. There were seven cases of adverse reactions in the placebo group, including nausea two cases, one case of insomnia, one case of fatigue, three cases of constipation. In Chen et al.’s (2020), one participant in the CHM group reported slight diarrhea, one participant reported mild constipation, and one participant had abnormal liver function (ALT, 108.7 U/L; AST, 63.7 U/L); no specific adverse reactions were reported in the placebo group. The comprehensive results showed that the incidence of adverse reactions was 4.68% in the CHM group and 3.44% in the placebo group, with no significant difference between the two groups [MD = 1.14; 95% CI (0.53, 2.42); *Z* = 0.33; *P* = 0.74] (Figure 11).

##### Chinese Herbal Medicine vs. Western Medicine Group

**FIGURE 12:**
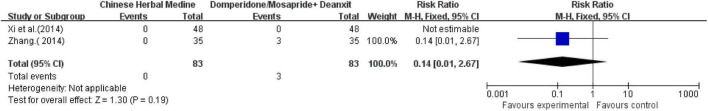
Meta-analysis of adverse reactions of CHM vs. western medicine group.

Out of the six studies (Xi et al., 2014; Zhang, 2014; Zhang et al., 2016, 2017a,b,c; Li et al., 2018), four conducted drug safety evaluation. Among them Zhang et al. (2016, 2017a,b,c) mentioned only in the abstract that there were no adverse reactions in either group during the study period. Li et al. (2018) conducted a 6-month follow-up, and the result was that there were no serious adverse reactions in both groups. During Zhang (2014) treatment, two patients in the control group had mild dizziness, one patient had dry mouth and slight bitterness, and no adverse reactions occurred in the treatment group. During the Xi et al. (2014) study, no adverse reactions occurred in both groups. None of the reported adverse reactions affected the course of treatment. The comprehensive results showed that there was no significant difference in the occurrence of adverse reactions between the two groups [MD = 0.14; 95% CI (0.01, 2.67); *Z* = 1.30; *P* = 0.19] (Figure 12).

## Discussion

Our study suggests that herbal treatment of FD with psychological disorders has great potential to improve both dyspeptic symptoms and anxiety/depressive states with good clinical safety. To our knowledge, no other study has done a systematic review and meta-analysis of any treatment for FD with psychological disorders. Psychological factors are not only important in the development of the FD, but also have an important impact on the prognosis and quality of life of patients. Studies have confirmed (Lin et al., 2019; Esterita et al., 2021) that FD is significantly associated with depression and anxiety disorders. The improvement of anxiety and sleep disorders contributes to sustained remission of FD symptoms over a period of 3–6 months (Singh et al., 2021). Clinical consideration of psychological factors may contribute to better management of FD.

Previous meta-analyses have shown that CHM is significantly better than placebo in improving global symptoms of dyspepsia (Yu et al., 2016; Ho et al., 2022). Our study on the efficacy of CHM vs. placebo in the treatment of FD with psychological disorders showed better results than placebo in terms of total effective rate, total syndrome score, and depression scale. However, there was a high heterogeneity of the findings in all these aspects. This may be related to the inconsistency of the scales of assessment used in these aspects in the included studies.

Our study further compared the efficacy of CHM with that of positive medicine in the treatment of FD with psychological disorders. The results showed that CHM was more beneficial in the total effective rate, depression scale, HAMA score, and gastric emptying rate compared to the mosapride/dopantelone + deanxit group. Currently, pharmacologic treatment is the main treatment for FD, and for patients with psychological disorders, psychotropic treatments are generally selected. However, due to the complexity of the FD mechanism, the heterogeneity of the resulting symptoms, and the fact that the same symptoms may be caused by different etiologies, there is no uniform therapeutic drug in clinical practice. This is the reason why the selection of effective control drugs was more difficult in our study. During the screening of the literature, it was found that researchers chose a wide variety of control drugs, including those using only prokinetics or acid inhibitors or psychotropic drugs, as well as multiple drug combinations. The combination of prokinetics with deanxit was the most studied, so we used this as a control group.

One meta-analysis study found that there was no significant difference in the therapeutic effect of the prokinetics domperidone and mosapride in FD, so we combined the studies of domperidone + deanxit with mosapride + deanxit for analysis (Yang et al., 2017). In China, deanxit and fluoxetine were the most common anti-depressant drugs used for FD based on a study in 2019 (Luo et al., 2019). The efficacy of flupenthixol melitracen on overall FD symptoms and on depression and anxiety in patients with chronic somatic diseases has been demonstrated (Hashash et al., 2008; Wang et al., 2015). Some studies have shown that CHM is more effective than prokinetics in relieving global dyspeptic symptoms (Chu et al., 2018; Ho et al., 2021). Our study provides a stronger foundation for the effectiveness of CHM in treating FD with psychological disorders by comparing it with positive western medicine. However, there was high heterogeneity in HAMA, HAMD scores, and gastric emptying rate among the studies. The studies included different levels of psychological disorders, so the baseline HAMA and HAMD scores were inconsistent across studies. In addition, differences in Chinese herbal prescriptions may have led to differences in the degree of efficacy. The write-up of reasons may explain the high heterogeneity of HAMA and HAMD scores. Xi et al. (2014) did not introduce the test method of gastric emptying rate, and the high heterogeneity of gastric emptying rate still cannot be clearly explained.

Furthermore, none of the medical treatments are proven to alter the long-term natural history of FD (Ford et al., 2020). But four inclusion trials (Du et al., 2014; Xi et al., 2014; Zhang, 2014; Chen et al., 2020) reported better follow-up results of CHM in our study. Du followed up for 6 months, and there were five cases of recurrence in the placebo group. Chen conducted a 4-week follow-up and found that six patients in the placebo group relapsed and six patients in the CHM group had no recurrence. Xi conducted a 6-month follow-up, six cases recurred in the western medicine group and no cases recurred in the CHM group. Zhang conducted a 3-month follow-up and found that HAMD and gastric emptying were better improved in Zhang, who conducted a 3-month follow-up and found that HAMD and gastric emptying were better improved in the CHM group.

In this meta-analysis, adverse reactions were not mentioned in 2 of the 11 studies (Han and Wang, 2011; Lu and Chen, 2011). In the remaining studies, no serious adverse reactions were found in the CHM group for FD with psychological disorders. A meta-analysis result of Ford et al. (2017) showed that the total numbers of adverse events, and the adverse events leading to withdrawal in the psychotropic drugs group were significantly more common than those in the placebo group. In our study, there was no significant difference in the incidence of adverse reactions compared with the placebo or western medicine groups. It may be related to the good safety of deanxit applied within 2 weeks (Luo et al., 2019). Previous clinical studies of CHM for the treatment of FD have also shown a high level of safety (Yu et al., 2016; Chu et al., 2018; Gwee et al., 2021; Ho et al., 2021, 2022).

In conclusion, this study collected clinical data from RCTs and evaluated systematically and objectively the clinical efficacy and safety of CHM for FD with psychological disorders treatment using evidence-based medicine. It provides evidence for the efficacy and safety of CHM in the treatment of FD with psychological disorders and suggests that CHM has great potential in the clinical application of this disease. Also, in agreement with Gwee et al. (2021), an attractive aspect of herbal medicine is the prospect of targeting multiple pathophysiological mechanisms simultaneously. In-depth mechanistic studies should be followed up.

## Limitations

In this study, there are still some deficiencies. Most of the included studies were completed in China, and the statistical results have certain regional characteristics. The number of included studies and subjects was small, and there were differences in the prescriptions and dosage forms of the included CHM. Due to the different prescriptions of the herbal treatment group, all 11 studies were not combined for analysis but were divided into two parts according to the difference of the control group (placebo group, prokinetics + deanxit group). This approach, while attempting to avoid higher heterogeneity, results in no more than six items in each part, which hinders funnel plot analysis and publication bias detection.

## Data Availability Statement

The raw data supporting the conclusions of this article will be made available by the authors, without undue reservation.

## Author Contributions

WW was responsible for the design and conception of this study. XL and LW conducted the statistical analysis, graph drawing, and manuscript writing. SF, XQ, TJ, XS, and YY were responsible for searching databases, screening documents, extracting data, and evaluating methodological quality. All authors critically revised the manuscript.

## Conflict of Interest

The authors declare that the research was conducted in the absence of any commercial or financial relationships that could be construed as a potential conflict of interest.

## Publisher’s Note

All claims expressed in this article are solely those of the authors and do not necessarily represent those of their affiliated organizations, or those of the publisher, the editors and the reviewers. Any product that may be evaluated in this article, or claim that may be made by its manufacturer, is not guaranteed or endorsed by the publisher.
